# Diagnosis of a hepatic hydatid cyst using posteroanterior chest radiography

**DOI:** 10.1590/0037-8682-0205-2022

**Published:** 2022-07-25

**Authors:** Yener Aydin, Ali Bilal Ulas, Atilla Eroglu

**Affiliations:** 1 Ataturk University, Medical Faculty, Department of Thoracic Surgery, Erzurum, Turkey.

A 64-year-old male patient with chronic obstructive pulmonary disease presented to our hospital with back pain. Posteroanterior chest radiography findings indicated a calcified, solid nodule having smooth borders and located on the right side, under the diaphragm (Figure 1). Based on the findings, a presumptive diagnosis of a hepatic hydatid cyst was made.

**FİGURE 1:**
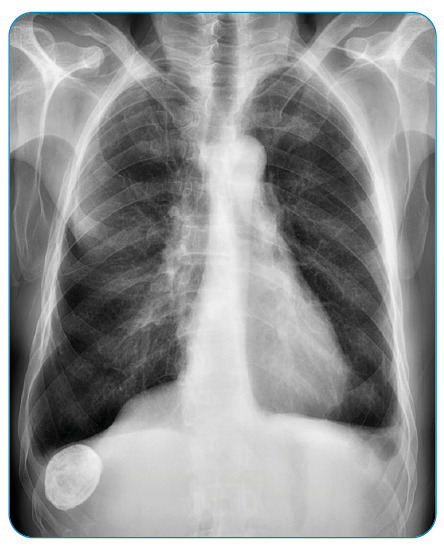
Posteroanterior chest radiography showing a subdiaphragmatic calcified hepatic hydatid cyst.

A hydatid cyst is a parasitic disease caused by *Echinococcus granulosus*. It is a notable health problem in regions where livestock is still widely dealt with^1^. The liver and the lungs are the affected organs in approximately 80% and 22% of cases, respectively^2^. When a hydatid cyst is ruptured and complicated, the patient may have several additional radiological findings^3^. Malignant diseases and other cystic lesions should be considered when making the differential diagnosis. Ultrasonography, computed tomography, magnetic resonance imaging, and serological tests can be performed as complementary techniques for diagnosis^3^. Our findings suggest that a calcified hepatic hydatid cyst can sometimes be detected through direct chest radiography.
